# Nursery Application of Raw and Thermally Treated *Hermetia illucens* Frass Shows Dose-Dependent Effects Against *Fusarium oxysporum* f. sp. *lycopersici* on Tomato Under Greenhouse Conditions

**DOI:** 10.3390/insects17070669

**Published:** 2026-06-26

**Authors:** Luca Alfarano, Sara Bellezza Oddon, Riccardo Cecire, Laura Gasco, Massimo Pugliese

**Affiliations:** 1 Department of Agricultural, Forest and Food Sciences (DISAFA), University of Torino, 10095 Grugliasco, Italy; sara.bellezzaoddon@unito.it (S.B.O.); riccardo.cecire@unito.it (R.C.); laura.gasco@unito.it (L.G.); massimo.pugliese@unito.it (M.P.); 2 Interdepartmental Centre for Innovation in the Agro-Environmental Sector, AGROINNOVA, University of Torino, Largo Braccini 2, 10095 Grugliasco, Italy

**Keywords:** frass, *Hermetia illucens*, *Fusarium oxysporum* f. sp. *lycopersici*, tomato, disease, biological control, soil amendment, upcycling, plant growth promotion, Fusarium wilt

## Abstract

Interest in the rearing of *Hermetia illucens* L. by-products has increased in recent years. This study evaluated the effects of *H. illucens* frass, obtained from larvae reared on a Gainesville diet, for the control of *Fusarium oxysporum* f. sp. *lycopersici* (Sacc.) W.C. Snyder & H.N. Hansen (FOL). Greenhouse trials were conducted comparing thermally treated (70 °C for 60 min) and untreated frass applied at different concentrations (1–20% *v*/*v*). Disease severity, disease incidence, area under the disease progress curve (AUDPC), and plant fresh weight were assessed. Frass application significantly reduced disease severity and incidence, compared with the inoculated untreated control, with the greatest reductions observed in the 20% thermally untreated treatment and in the 5% and 10% thermally treated treatments under the greenhouse conditions of this study. The area under the disease progress curve (AUDPC) analysis suggested a reduction in disease progression over time. No significant differences were observed between thermally treated and untreated frass. Fresh weight was mainly influenced by frass dose, and the highest values were obtained with treatments at 10% and 20%. *H. illucens* frass showed preliminary potential as a sustainable starter for the suppression of Fusarium wilt of tomato under greenhouse conditions.

## Simple Summary

Tomatoes are affected by different soil-borne diseases, in particular Fusarium wilt that can cause important yield losses. Chemical products, where available, are mainly used to control this disease. With the aim of studying new alternative natural substances to reduce the chemical inputs in agriculture, we applied frass, a natural amendment produced by black soldier fly larvae, at 1–20% (*v*/*v*), to growing media before seeding and tested on potted tomato plants in greenhouse. Frass applied in the nursery phase reduced disease symptoms, with a suppression of 88% observed in the thermally untreated treatment at the highest concentration tested, (20% *v*/*v*), and 79% and 64% for frass thermally treated at 5% (*v*/*v*) and 10% (*v*/*v*), respectively, under greenhouse conditions. Thermal treatment did not significantly change disease suppression under the tested conditions. Nevertheless, this comparison does not allow for definitive conclusions on the underlying mechanisms. Our results suggest that insect-derived frass can be valorized as a suppressive amendment and may represent a complementary tool for reducing Fusarium wilt disease on tomato, while also providing information on dose–response effects and potential phytotoxicity.

## 1. Introduction

Tomato (*Solanum lycopersicum* L.) represents one of the most widely cultivated horticultural crops, playing a key role in both the fresh market and processing industries. According to FAOSTAT production data [1], global annual production reached about 188 million tons in 2024. Italy is approximately the sixth largest tomato producer worldwide and among the leading producers in the Mediterranean region, with a harvested area of about 102,800 ha [1]. Despite the high global production of tomato, crop yields are strongly affected by plant pathogens and pests, which are estimated to cause worldwide losses ranging from 20 to 40% across major food crops [2].

An important soil-borne pathogen associated with tomato is the ascomycete fungus *Fusarium oxysporum* f. sp. *lycopersici* (Sacc.) W.C. Snyder & H.N. Hansen (FOL), the causal agent of vascular wilt in tomato, belonging to the *Fusarium oxysporum* Schltdl. species complex, which includes numerous formae speciales with distinct host specificity [3]. FOL causes tomato Fusarium wilt, with symptoms resembling water stress, such as leaf yellowing and general wilting [4,5], often resulting in severe yield losses in different areas of the world [6,7].

Environmental conditions are known to modulate disease expression and can consequently influence variability in yield reductions. Environmental and soil related factors can affect pathogen survival, root infection and disease development [8], while high temperatures may increase disease severity [5]. Under favorable environmental conditions (27–30 °C), FOL can cause yield losses of 45–55%, increasing to approximately 70% during severe outbreaks [9]. In line with these observations, severe outbreaks of Fusarium wilt have been reported in several tomato growing regions around the world. In Mexico, field surveys reported disease incidence ranging from 10 to 85% [10]. In Italy, studies conducted during the 1990s reported a mortality rate of up to 60% during severe outbreaks [11]. Overall, these data highlight the relevance of Fusarium wilt as a major constraint to tomato production.

The chemical management of FOL has progressively declined over the last decades due to the restriction of several fumigants and fungicides because of their environmental and toxicological impacts. Currently, only a limited number of fumigants remain available in the EU, including metam sodium, metam potassium and dazomet. Methyl bromide, previously one of the most widely used fumigants for soil-borne pathogens, was phased out worldwide following the Montreal Protocol restrictions [12,13]. Consequently, increasing attention has been devoted to alternative approaches, including resistant cultivars, biological control agents (BCAs) and organic amendments [14].

The management of Fusarium wilt depends on the agricultural system adopted, whether conventional, integrated or organic. It relies on a combination of good agricultural practices, including the removal of infected plants, prevention of water stagnation, the use of physical methods such as steam treatment or soil solarization, crop rotation, resistant varieties and grafting [5,8], application of elicitor substances such as salicylic acid and humic acids [15], and organic amendments capable of promoting soil suppressiveness against *F. oxysporum* [16,17].

In recent years, considerable attention has been devoted to the use of BCAs [18,19], particularly *Bacillus* spp. and *Trichoderma* spp., which are among the most widely investigated microbial antagonists [19,20].

Integrative approaches combining BCAs with organic amendments such as manure, compost and plant residues have been explored as a strategy to suppress Fusarium wilt in tomato [21,22,23]. The use of organic amendments is also encouraged by the European policy programs.

In line with the 2030 Agenda and circular economy strategies, scientific interest in *Hermetia illucens* L. (HI) has increased over time, both for the use of larvae in animal feed [24,25,26] and for applications in waste bioconversion [27,28,29,30].

The remaining material, frass, obtained during the bioconversion of organic substrate by HI, consisting of a mixture of excrement derived from farmed larvae, residual feeding substrate, parts of farmed insects (exuviae), and dead eggs, has chemical characteristics, composition and properties that make it a promising soil amendment for agricultural applications [31,32,33,34,35,36].

It is rich in organic matter and contains valuable nutrients for soil health, such as nitrogen, phosphorus, and potassium. Moreover, frass includes insect exuviae (shed exoskeletons), which are a significant source of chitin [37], a compound known for its beneficial effects on soil quality and plant resilience and reported to act as an elicitor of plant defense responses [38,39].

In addition, frass contains other biostimulant components such as amino acids, humic substances, phytohormones, and beneficial microbes [40,41].

Frass from HI improves soil fertility and may exert a biostimulant effect, the latter manifesting in a stronger natural response from plants subjected to fungal attack [32]. Antifungal properties were also shown in vitro against Basidiomycota, Oomycetes, and Ascomycota fungi such as *Alternaria solani* Kühn, *Botrytis cinerea* Pers., *Sclerotinia sclerotiorum* (Lib.) de Bary, and *F. oxysporum* [37]; these effects have been attributed to the presence of *Bacillus velezensis* Ruiz-García et al. and the production of antimicrobial compounds such as fengycins and iturins.

However, although previous studies have demonstrated the general suppressive potential of HI frass against soil-borne pathogens [42], important aspects related to its practical application during nursery production remain insufficiently explored. In studies involving suppressive organic amendments, substrate applications performed during the early stages of plant growth were shown to reduce Fusarium wilt development during subsequent plant growth under controlled conditions [21].

Therefore, the present study also investigated whether HI frass applied at sowing could provide similar protective effects against FOL during the post-transplant growth stages.

Furthermore, the effect of application rate on disease suppression has not been consistently addressed in the literature, and it is unclear whether increasing frass concentration leads to proportional improvements in efficacy or may result in diminishing returns or phytotoxic effects.

From an applied perspective, defining the response to different application rates is essential to identify effective and safe dosage ranges for subsequent experimental comparisons, including studies involving different substrates, diets, or integrated management strategies. The dose optimization also represents a critical factor in agronomic contexts, where the economic cost of amendments and the risk of phytotoxic effects directly influence their practical feasibility and adoption.

In addition, the influence of thermal treatment on frass efficacy remains uncertain, especially in relation to its compatibility with regulatory requirements for the commercialization of insect-derived by-products.

It is still unclear whether disease suppression mainly depends on viable microbial populations associated with frass or heat-stable compounds naturally present in the material [21,43]. However, the relative contribution of these factors has not been directly investigated in most applied studies and remains difficult to define under greenhouse conditions.

Therefore, the aim of this study was to evaluate the effect of thermally treated and untreated HI frass applied before seeding at increasing concentrations on the management of FOL in tomato after transplanting. This study should be considered a greenhouse-based, short-term, single-pathosystem experimental investigation aimed at exploring nursery application, dose-dependent response and the influence of thermal treatment on FOL control under controlled conditions. Within this context, the study aimed to explore whether frass efficacy followed a dose-dependent response and whether increasing concentrations influenced both disease suppression and plant fresh weight, including the identification of a potential threshold associated with phytotoxic effects. Therefore, this work extends existing literature by providing empirical validation of application parameters, relevant for the practical use of HI frass in tomato to suppress FOL.

## 2. Materials and Methods

### 2.1. Frass Production and Heat Treatment

*H. illucens* frass was produced at the experimental center Tetto Frati of the University of Turin (Carmagnola, Turin, Italy).

Reproduction of HI adults was carried out in a climatic chamber using a Dark-Love cage system, maintained at 30 °C and 70% relative humidity. Adult emergence was synchronized, and egg collection was performed twice per week at 24 h intervals. Larvae hatched without substrate and were collected every 24 h (1-day-old larvae). After collection, they were inoculated onto Gainesville diet [44].

At 6 days of age, larvae were estimated and transferred to fattening boxes, which were kept in a climatic chamber at 28 °C and 60% relative humidity. Larvae were reared from day 6 to day 12 at a feeding rate of 0.7 g of wet Gainesville diet per larva. At 12 days of age, larvae were harvested by sieving (4 mm mesh) and separated from the frass. The frass was stored at environmental temperature in closed bags, and part was thermally treated at 70 °C for 60 min, using a dryer (B.MASTER, Tauro Essiccatori S.r.l., Cassiano Vicentino, Italy). The same heat-treatment protocol was applied to all frass batches, as all treated quantities were within the operational loading capacity of the dryer (100 kg), allowing the same treatment conditions to be applied across batches. The heat treatment was applied to obtain a product compliant with Commission Regulation (EU) No. 142/2011, concerning the treatment of animal by-products for use as fertilizers.

### 2.2. Chemical Composition and Physicochemical Properties of Frass

The chemical composition and physicochemical properties of frass were determined at the agricultural chemistry laboratory of the University of Turin on two types of samples: thermally treated and untreated frass to evaluate the effect of heat treatment. Dry matter content was determined by drying the samples at 105 °C. Total nitrogen was determined using the Kjeldahl method.

pH measurements were performed using a potentiometric pH meter (CyberScan pH 510 ion meter, Eutech Instruments Pte Ltd., Singapore) while electrical conductivity was assessed with a conductivity meter (Cond 51+ conductivity meter, XS Instruments S.r.l., Carpi, Italy).

Total inorganic elements were determined after sample pre-treatment with a muffle furnace at 450 °C for 4 h, followed by acid digestion with 10 mL of 1 N HCl and 0.5 mL of 1 N HNO_3_ on a hot plate at approximately 150 °C for 2 h. The digested samples were filtered and brought to volume (50 mL) flasks, and elemental concentrations were quantified by inductively coupled plasma optical emission spectroscopy (ICP-OES) (iCAP PRO ICP-OES system, Thermo Fisher Scientific, Waltham, MA, USA). All analyses were performed in triplicate (analytical replicates).

### 2.3. Production and Inoculation of the Pathogen

A highly virulent strain of FOL belonging to race 1 (Agroinnova collection, University of Turin, Grugliasco, Italy), maintained as frozen stocks at −80 °C in potato dextrose agar, was used in this study.

The mycelium of this ascomycete fungus was cultivated on potato dextrose agar (PDA) at room temperature (25 ± 2 °C).

When the mycelium achieved complete and homogeneous colonization of the PDA plates, mycelial plugs were transferred onto fresh PDA plates under sterile conditions to obtain a uniform and clean culture. After sufficient growth was achieved, fungal inoculum was prepared by transferring agar plugs containing actively growing mycelium into a 1 L flask containing 200 g of wheat kernels previously sterilized by autoclaving at 121 °C for 30 min.

All operations were performed under a laminar flow cabinet to avoid contamination. One small fungal plug (5 mm in diameter) was excised and inserted into each flask, which was incubated for 10 days. The colonized wheat kernels were used as inoculum and mixed with the substrate prior to transplanting. Inoculated pots received FOL at a rate of 1 g L^−1^ of substrate in 2 L pots. The inoculation was performed 7 days before transplanting seedlings from the nursery to the pots. The inoculum density (1 g L^−1^ of substrate) was selected to ensure uniform and reproducible disease pressure under greenhouse conditions, allowing the detection of differences among treatments without causing excessively rapid plant collapse. Similar inoculum levels have previously been used in greenhouse trials on Fusarium wilt of tomato and other soil-borne pathogens in potted crops [45,46].

### 2.4. Frass Application and Experimental Design

The two trials were conducted in Agroinnova greenhouses. Plants were grown under controlled environment conditions, with a temperature of 24–30 °C and relative humidity of 65–75%, around the growth optimum of the FOL-tomato pathosystem.

Frass (thermally treated and untreated) was applied at different volumetric concentrations (*v*/*v*), by mixing it with the growth substrate (TS3 + Cyclamen, 50% + 50%, Turco Silvestro, Bagnasco, Italy), in nursery pots prior to transplanting. The volumes required for each treatment were calculated according to the target volumetric percentage. The experimental design included different concentrations of thermally treated frass (1%, 5%, 10%, corresponding to 2.1, 10.6, and 21.2 g/L, respectively) and thermally untreated frass (1%, 5%, 10%, 20%, corresponding to 1.8, 9.2, 18.3 and 36.6 g/L, respectively). The selected dose levels were chosen to represent a gradient from low to relatively high amendment rates, in order to evaluate a response trend across increasing concentrations rather than to define optimal application rates. This range was selected to capture potential threshold effects, including both sub-optimal responses at low doses and possible phytotoxic effects at higher concentrations. Similar application ranges have been reported for organic amendments in greenhouse experiments, where concentrations up to 20% (*v*/*v*) have been assayed [47]. In addition, a previous study on HI frass reported positive effects up to 20% (*v*/*v*), whereas higher application rates (>20%) were associated with reduced crop performance and potential phytotoxic effects [48].

A fungicide treatment, a non-inoculated control and an inoculated untreated control were included for comparison. The chemical control consisted of thiophanate-methyl (41.7 g, 500 g L^−1^, Enovit Metil^®^ FL, Sipcam OXON S.p.A., Milan, Italy). Two applications were performed: after transplanting (0.14 mL L^−1^) and after 7 days (0.5 mL L^−1^).

Tomato seeds (*S. lycopersicum*, lot 000067266, Four) were sown in trays containing sterile substrate and maintained under controlled greenhouse conditions. After seedling establishment, plants were transplanted into 2 L pots previously filled with the treated substrates. The pots were watered daily.

This experimental scheme was adopted according to a completely randomized design, for both thermally treated and thermally untreated frass. Each pot represented one experimental replicate and contained two tomato plants. Each treatment included five inoculated and five uninoculated pots, corresponding to a total of 10 plants per treatment.

### 2.5. Disease and Plant Growth Evaluation

Indicators of plant growth and disease development were monitored throughout the experimental period, until 42 days after inoculation. Disease severity (DS) and disease incidence (DI) were assessed throughout the experimental period by visual observation of symptoms typical of Fusarium wilt, including leaf yellowing, wilting and vascular browning.

Assessments were conducted starting from the early germination stages in the nursery, to evaluate plant growth, including leaf number, plant height and germination rate, in each nursery pot. This approach allowed the detection of potential phytotoxicity effects associated with the different concentrations provided.

After transplanting, at the end of the trial (42 days after inoculation), disease severity (DS) and disease incidence (DI) were assessed by longitudinally cutting stems and evaluating vascular browning/discoloration. Disease severity was scored according to a 0–4 scale adapted from the vascular discoloration assessment approach described in [49], as follows: 0 = healthy, no vascular browning; 1 = initial vascular browning, with slight symptom expression; 2 = moderate vascular browning, associated with initial wilting symptoms; 3 = severe vascular browning, associated with severe wilting and growth reduction; and 4 = complete vascular browning, associated with plant death. Scores from 1 onward were considered indicative of vascular browning symptoms. Plant growth was assessed by measuring above-ground fresh weight. Shoots were cut at the substrate surface and immediately weighed. Fresh weight was recorded for each plant and used as an indicator of plant growth response under the experimental condition.

For statistical analysis and graphical representation, disease severity was first scored on the 0–4 scale described above and then converted into a percentage using the following formula:

DS% = (mean disease severity score × 100)/4.

Disease incidence (DI) was calculated as the percentage of symptomatic plants relative to the total number of assessed plants, using the following formula [50]:

DI% = (number of symptomatic plants)/total number of plants) × 100.

Based on three disease severity assessments, the area under the disease progress curve (AUDPC) was calculated using the trapezoidal integration method [51]:

$$AUDPC=\sum_{i=1}^{n-1}\frac{\left(y_{i}+y_{i+1}\right)}{2}(t_{i+1}-t_{i})$$

where *y_i_* is the disease severity at the *i*th assessment, *t_i_* is the time (days) at the *i*th observation, and *n* is the total number of disease assessments. AUDPC values were expressed as disease severity per day.

### 2.6. Statistical Analysis

Before pooling the data from the first and second trials, a factorial ANOVA was performed including trial, frass dose and thermal treatment as fixed factors, in order to evaluate the presence of trial effects and their interactions with the experimental factors. Since no significant effects of trial or its interaction were detected (*p* > 0.05), as shown in Supplementary Table S1, data from both trials were pooled for the analyses of disease severity (DS), disease incidence (DI), AUDPC and above-ground fresh weight.

Statistical analyses were performed using IBM SPSS Statistics for Windows, Version 26.0 (IBM Corp., Armonk, NY, USA). Homogeneity of variance and heteroscedasticity were assessed before performing ANOVA using Levene’s test and the F-test, respectively. When necessary, data were transformed using Tukey’s ladder of powers to satisfy the assumptions of parametric analysis. In particular, disease severity (%) data were arcsin square-root transformed for both one-way and two-way ANOVA, while AUDPC values were square-root transformed for the two-way ANOVA. However, figures and tables report non-transformed means to facilitate interpretation of the results.

When assumptions of homogeneity of variance and absence of heteroscedasticity were satisfied (*p* > 0.05), data were analyzed by one-way ANOVA followed by Tukey’s HSD post hoc test (*p* < 0.05). For AUDPC, Welch’s ANOVA followed by the Games–Howell post hoc test was applied because the assumptions of homogeneity of variances and absence of heteroscedasticity were not satisfied. Two-way ANOVA analyses were also conducted to evaluate the effect of frass dose, thermal treatment and their interaction on disease severity (%), disease incidence (%) and fresh weight.

## 3. Results

**Figure 1:**
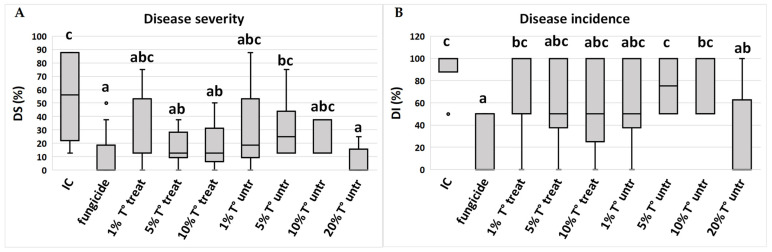
Disease severity (%) (**A**) and disease incidence (%) (**B**) of tomato plants. Data from the first and second trials were pooled and analyzed together. Boxplots show median values, interquartile ranges and data dispersion. Different letters indicate significant differences according to one-way ANOVA, followed by Tukey’s post hoc test (*p* < 0.05). The overall ANOVA was significant for both disease severity (*p* < 0.001) and disease incidence (*p* < 0.001). Legends: IC = inoculated untreated control; fungicide = inoculated fungicide control; T° treat = inoculated thermally treated frass; T° untr = inoculated thermally untreated frass; circles represent outlier values.

**Figure 2:**
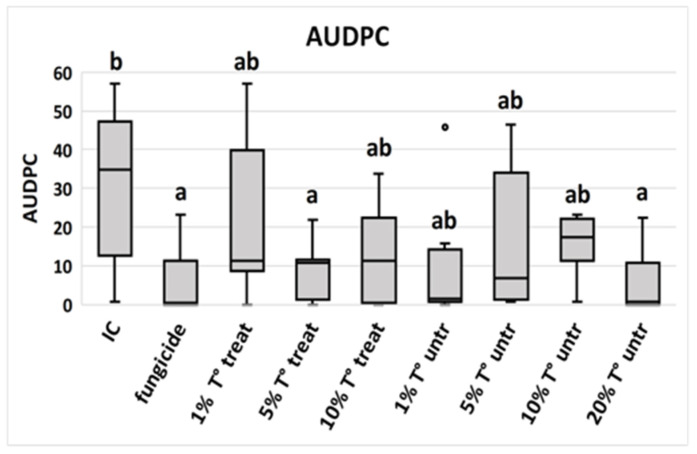
AUDPC of tomato plants. Data from the first and second trials were pooled and analyzed together. Boxplots show median values, interquartile ranges and data dispersion. Different letters indicate significant differences according to Welch’s ANOVA, followed by the Games–Howell post hoc test (*p* < 0.05). The overall Welch’s ANOVA was significant for AUDPC (*p* = 0.007). Legends: IC = inoculated untreated control; fungicide = inoculated fungicide control; T° treat = inoculated thermally treated frass; T° untr = inoculated thermally untreated frass; circles represent outlier values.

**Figure 3:**
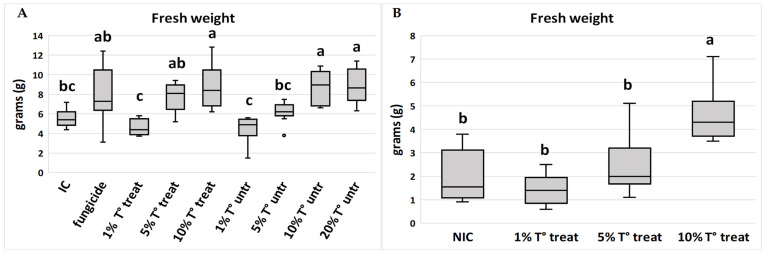
Above-ground fresh weight (g) (**A**) of tomato plants inoculated with *Fusarium oxysporum* f. sp. *lycopersici*. Above-ground fresh weight (g) (**B**) of tomato non-inoculated plants. Data from the first and second trials were pooled and analyzed together. Boxplots show median values, interquartile ranges and data dispersion. Different letters indicate significant differences according to one-way ANOVA, followed by Tukey’s post hoc test (*p* < 0.05). The overall ANOVA was significant for fresh weight in A and B (*p* < 0.001). Legends: NIC = non-inoculated untreated control; IC = inoculated untreated control; fungicide = inoculated fungicide control; T° treat = inoculated thermally treated frass; T° untr = inoculated thermally untreated frass; circles represent outlier values.

Disease severity, disease incidence, area under the disease progress curve (AUDPC) and fresh weight differed significantly among treatments (*p* < 0.05) (Figure 1, Figure 2 and Figure 3).

No significant effect of trial or its interaction with dose and thermal treatment was detected (*p* > 0.05; Supplementary Table S1), indicating consistency between experiments and supporting data pooling.

The treatment with thermally treated frass at 20% (*v*/*v*) caused severe phytotoxic effects during the nursery phase, resulting in seedling death, and was therefore excluded from the subsequent analyses. This effect was not observed for thermally untreated frass at the same concentration.

### 3.1. Physicochemical Characterization of Thermally Treated and Untreated Frass

The chemical composition and physicochemical properties of thermally treated and untreated frass are reported in Table 1. The measured parameters include dry matter, pH, electrical conductivity, and nutrient contents (N, P, K). Analyses were performed in triplicate (analytical replicates).

### 3.2. Disease Severity and Incidence

The inoculated untreated control showed the highest disease severity and incidence, whereas the lowest disease severity was observed in plants treated with thermally untreated frass applied at 20% (*v*/*v*), with no significant differences from the fungicide treatment (Figure 1).

Thermally treated frass applied at 5% (*v*/*v*) and 10% (*v*/*v*) significantly reduced disease severity by 70% and 64%, respectively, compared to the inoculated untreated control, reaching levels that did not differ significantly from both the fungicide treatment (81% reduction) and the thermally untreated frass at 20% (*v*/*v*) (88% reduction) (Figure 1).

Disease incidence followed a similar pattern. In particular, thermally untreated frass at 20% (*v*/*v*) significantly reduced disease incidence by approximately 72% compared to the untreated control, showing values that did not differ significantly from the fungicide treatment, which showed an efficacy of approximately 83% (Figure 1).

Two-way ANOVA revealed a significant effect of frass dose on both disease severity and incidence, whereas thermal treatment and the interaction between dose and treatment were not significant (Table 2). In particular, the 20% dose significantly reduced disease severity and disease incidence compared to 1%, 5% and 10%, which did not differ from each other (Table 2).

The untreated control showed the highest AUDPC values, indicating the greatest disease progression. In contrast, fungicide treatment and 20% (*v*/*v*) thermally untreated frass respectively reduced AUDPC by 82% and 82%, compared to the inoculated untreated control (Figure 2). Similarly, 5% (*v*/*v*) thermally treated frass reduced AUDPC by 74% and did not differ significantly from the fungicide treatment or from 20% (*v*/*v*) thermally untreated frass. Boxplot distributions showed partial overlap among treatments associated with intermediate AUDPC reductions, whereas fungicide treatment, 5% (*v*/*v*) thermally treated frass and 20% (*v*/*v*) thermally untreated frass showed consistently lower AUDPC values (Figure 2).

### 3.3. Fresh Weight

Under pathogen pressure (Figure 3A), above-ground fresh weight also varied among treatments. The highest fresh weight values were observed in plants treated with thermally untreated frass applied at 10–20% (*v*/*v*), corresponding to increases of 58% and 60%, respectively, compared to the inoculated untreated control, with values that did not differ significantly from the fungicide treatment (increase of 41%) and from 10% (*v*/*v*) thermally treated frass. Similarly, 10% (*v*/*v*) thermally treated frass increased fresh weight by 58% compared to the inoculated untreated control. Boxplot distributions showed a wider dispersion of fresh weight values in treatments associated with the highest biomass accumulation, particularly fungicide and 10–20% (*v*/*v*) thermally untreated frass (Figure 3A). In contrast, lower fresh weight values were obtained from plants treated with lower frass concentrations, particularly 1% (*v*/*v*) thermally treated and thermally untreated frass. Above-ground fresh weight in non-inoculated plants (B) was significantly affected by frass application rate (Figure 3B). Plants treated with thermally treated frass applied at 10% (*v*/*v*) showed the highest fresh weight values, corresponding to an increase of 129% compared to the non-inoculated untreated control, and differed significantly from the other treatments. Boxplot distributions in non-inoculated plants confirmed the higher fresh weight values observed with 10% (*v*/*v*) thermally treated frass compared to the other treatments (Figure 3B). Two-way ANOVA revealed a significant effect of frass dose on above-ground fresh weight, whereas thermal treatment and the interaction between dose and treatment were not significant (Table 3). In particular, plants treated with 10% and 20% frass showed significantly higher fresh weight compared to 1% and 5%.

## 4. Discussion

In this experimental study, dose-dependent responses were assessed and the effects of HI frass, derived from larvae reared on a Gainesville diet, on FOL disease development and above-ground fresh weight of tomato plants were evaluated under controlled greenhouse conditions by applying this amendment to the substrate cultivation in nursery phase. Such conditions are particularly relevant for nursery production systems, where early-stage application of substances can influence plant health and disease development after transplanting. In this context, seed biopriming and treatments applied at the seedling stage have been shown to significantly reduce Fusarium wilt severity [52].

Among the assayed treatments, the 20% (*v*/*v*) dose of thermally untreated frass showed consistently low disease severity values, corresponding to an approximately 88% reduction compared to the inoculated untreated control, while disease incidence was reduced by approximately 72% at the same concentration. Under the described experimental greenhouse conditions, disease severity values obtained with this treatment did not differ significantly from those observed with the fungicide treatment, which reduced disease severity and incidence by approximately 81% and 83%, respectively. Similarly, 5% and 10% (*v*/*v*) thermally treated frass significantly reduced disease severity by approximately 70% and 64%, respectively. These treatments did not differ significantly from either the fungicide treatment or 20% (*v*/*v*) thermally untreated frass. This result indicates that disease suppression was influenced by frass concentration but did not follow a strictly linear dose-dependent pattern. The similar levels of disease control observed between 5% and 10% (*v*/*v*) thermally treated frass and 20% (*v*/*v*) thermally untreated frass further suggest the presence of a response plateau rather than a clear maximization at the highest dose, while still showing increased efficacy as the dose increased.

In contrast, reductions in disease incidence obtained with 5% and 10% (*v*/*v*) thermally treated frass were lower, corresponding to approximately 39% and 38%, respectively, suggesting that frass application at these doses affected symptom severity more consistently than symptom occurrence. A similar trend was observed for thermally untreated frass at lower concentrations, where 1% and 5% (*v*/*v*) reduced disease incidence by approximately 33% and 17%, respectively, further supporting the absence of a clearly separated response gradient across all treatments. Moreover, boxplot distributions showed partial overlap among the interquartile ranges of 5% and 10% (*v*/*v*) thermally treated frass and 1–10% (*v*/*v*) thermally untreated frass. This suggests limited differentiation among intermediate treatments despite significant overall effects. In contrast, the highest dose assayed, 20% (*v*/*v*) thermally untreated frass, showed a clearer separation from the intermediate treatments.

This pattern is consistent with previous studies on organic amendments, where disease suppression does not necessarily increase proportionally with the application rate of the treatments and may reach a plateau or decline at higher concentrations depending on the amendment and pathosystem [47]. In addition, the occurrence of phytotoxic effects in the thermally treated frass at 20% (*v*/*v*) further supports the existence of an upper threshold, beyond which increasing the application rate may not provide additional benefits and may negatively affect plant growth with development reduction and plant death. Overall, these results indicate that the 20% (*v*/*v*) thermally untreated frass was the most effective concentration under the specific greenhouse conditions tested, but should not be interpreted yet as an optimal dose, also because conversely the same concentration (20% *v*/*v*) thermally treated showed strong phytotoxicity.

Statistical analysis identified frass dose, rather than thermal treatment or its interaction with dose, as the main factor influencing disease suppression (Table 2). This pattern is consistent with previous observations reporting no major differences between pasteurized and non-pasteurized HI frass [42], and with evidence that pasteurized frass can retain suppressive activity against fungal pathogens after heat treatment [53]. Although thermal treatment at 70 C° for 60 min has been reported to substantially reduce microbial activity, microbial biomass, viable bacterial counts and to alter microbial community composition in HI frass [54], thermal treatment was not identified as a significant factor affecting disease suppression in the present study. This suggests that application rate played a greater role than thermal processing under the greenhouse conditions tested.

The AUDPC results further confirmed the ability of frass to limit disease progression over time, supporting the persistence of its suppressive effect over the time considered in this study. In particular, thermally treated frass at 5% (*v*/*v*) and thermally untreated frass at 20% (*v*/*v*) showed the strongest reduction in disease progression, reducing AUDPC by 74% and 82%, respectively, compared to the inoculated untreated control. While lower concentrations resulted in more variable effects, as indicated by the broader distribution patterns observed in the boxplots, this suggests that sufficient frass concentrations are required to achieve a more stable suppressive effect over time, limited to the duration of this greenhouse trial, in which the final assessment was conducted at 42 days after inoculation.

Plant fresh weight accumulation, assessed as above-ground fresh weight, suggested a positive effect of higher frass application rates.

In non-inoculated plants, the 10% (*v*/*v*) thermally treated frass treatment resulted in the highest fresh weight values, corresponding to an increase of 129% compared to the non-inoculated untreated control, suggesting a positive effect on fresh biomass accumulation at higher concentrations. Under pathogen pressure, above-ground fresh weight also varied among treatments. The higher application rates (10% and 20% *v*/*v* thermally untreated frass) maintained the fresh weight values, with respective increases of 58% and 60% compared to inoculated untreated control. These values did not differ significantly from those observed for the fungicide treatment (41%) or from 10% (*v*/*v*) thermally treated frass, which increased fresh weight by 58%, indicating that frass application did not compromise above-ground fresh weight while contributing to disease suppression. The variability observed with 10% and 20% (*v*/*v*) thermally untreated frass suggests a less uniform fresh weight response under higher frass application rates, despite the overall positive effect on above-ground fresh weight accumulation. The physicochemical composition of thermally treated and untreated frass is reported in Table 1, showing alkaline pH values and the presence of relevant nutrient contents (N, P and K) in both frass types. However, no further interpretation was performed due to the descriptive nature of these analyses. The relationship between frass composition and plant growth responses therefore remains unclear and deserves further investigation.

The combination of nutritional and biostimulant properties of frass, including organic substances [42], chitin and chitosan [37], and other biostimulants such as amino acids, humic substances, phytohormones, and beneficial microbes [41], may have contributed to observed plant responses. However, the relative importance of these factors remains unclear. Their contribution cannot be distinguished based on the present experimental design because no microbiological analyses were performed to detect changes in soil microbial communities or to elucidate the mechanisms underlying disease suppression. Finally, it is important to acknowledge other limitations of this study. The experiments were conducted under controlled greenhouse conditions, which may not fully represent field environments. Plant growth was assessed using above-ground fresh weight as a comparative indicator under uniform greenhouse and irrigation conditions. Nevertheless, dry weight measurements could provide complementary information and should be included in future studies.

Future research should address these aspects and validate the effectiveness of HI frass under field conditions. Therefore, the present results should be considered preliminary evidence obtained under controlled greenhouse conditions, requiring validation across additional pathosystems and field environments.

## 5. Conclusions

This study investigated the effect of HI frass application rate and thermal treatment on the control of Fusarium wilt in tomato under controlled greenhouse conditions with application in nursery. The results indicate that application rate is a key factor influencing frass performance, although disease suppression did not follow a strictly linear dose–response pattern. While increasing application rates generally improved efficacy, excessively high concentrations may lead to phytotoxic effects, highlighting the need to identify application thresholds that balance disease control and plant performance.

From an applied perspective, the observation that thermal treatment did not significantly affect suppressive efficacy is particularly relevant. This suggests that regulatory sanitation requirements for insect derived by-products may be compatible with the preservation of frass functionality, supporting the practical use of HI frass within sustainable crop protection strategies and circular economy approaches.

However, these findings were obtained in a single greenhouse pathosystem and require validation under field conditions and across additional host–pathogen systems. Further studies are necessary to clarify the mechanisms of action and to evaluate the treatment under open-field conditions and on different cultivars and cultivation systems.

## Figures and Tables

**Table 1:** Physicochemical analysis of untreated and thermally treated frass.

	DM (%)	pH	Cond (mS)
Thermally treated frass	71	8.48	4.04
Thermally untreated frass	63	8.35	4.30
	**N (g/kg)**	**P (g/kg)**	**K (g/kg)**
Thermally treated frass	27.76	17.16	29.1
Thermally untreated frass	35.45	14.75	27.5

Chemical composition and physicochemical properties of thermally treated and untreated frass. Values represent the mean of three analytical measurements performed on the same sample. Legends: DM = Dry matter; Cond = electrical conductivity; N = nitrogen; P = phosphorus; K = potassium.

**Table 2:** Effects of frass dose, thermal treatment and their interaction on disease severity (%) and disease incidence (%) of tomato plants.

Factor	Level	Disease Severity% ± SD	Tukey	Significance
Frass Dose (% *v*/*v*)	1%	28.13 ± 26.55	b	*p* = 0.002
	5%	24.38 ± 19.22	b	
	10%	20.40 ± 13.95	b	
	20%	6.25 ± 10.62	a	
Thermal treatment	Treated	–	–	ns (*p* = 0.163)
	Untreated	–	–	
Dose × Thermal treatment	Interaction	–	–	ns (*p* = 0.377)
Frass Dose (% *v*/*v*)	1%	62.5 ± 35.82	b	*p* = 0.019
	5%	65 ± 32.85	b	
	10%	60.53 ± 31.53	b	
	20%	25 ± 42.49	a	
Thermal treatment	Treated	–	–	ns (*p* = 0.377)
	Untreated	–	–	
Dose × Thermal treatment	Interaction	–	–	ns (*p* = 0.532)

Data from the first and second trials were pooled and analyzed together. Values are reported as mean ± standard deviation (SD). Different letters indicate significant differences among dose levels according to two-way ANOVA, followed by Tukey’s post hoc test (*p* < 0.05). Legends: “–” = indicates that no values are reported for non-significant factors; ns = not significant.

**Table 3:** Effects of frass dose, thermal treatment and their interaction on above-ground fresh weight (g) of inoculated tomato plants.

Factor	Level	Fresh Weight (g) ± SD	Tukey	Significance
Frass Dose (% *v*/*v*)	1%	4.48 ± 1.06	c	*p* < 0.001
	5%	6.94 ± 1.48	b	
	10%	8.76 ± 1.82	a	
	20%	8.86 ± 1.79	a	
Thermal treatment	Treated	–	–	ns (*p* = 0.123)
	Untreated	–	–	
Dose × Thermal treatment	Interaction	–	–	ns *p* = 0.247)

Data from the first and second trials were pooled and analyzed together. Values are reported as mean ± standard deviation (SD). Different letters indicate significant differences among dose levels according to two-way ANOVA, followed by Tukey’s post hoc test (*p* < 0.05). Legends: “–” = indicates that no values are reported for non-significant factors; ns = not significant.

## Data Availability

The data that support the findings of this study are available from the corresponding author, Luca Alfarano, upon reasonable request.

## Supplementary Materials

The following supporting information can be downloaded at: https://www.mdpi.com/article/10.3390/insects17070669/s1, Table S1: Factorial ANOVA on arcsine-square-root transformed disease severity data showing consistency between two independent greenhouse trials.
