# Supplementary material for: Nursery Application of Raw and Thermally Treated Hermetia illucens Frass Shows Dose-Dependent Effects Against Fusarium oxysporum f. sp. lycopersici on Tomato Under Greenhouse Conditions
Source: Insects. 2026 Jun 26;17(7):669. doi: 10.3390/insects17070669 (PMC13411600; doi:10.3390/insects17070669)
Supplement: Supplementary file 1 [file insects-17-00669-s001.zip › insects-4258996-supplementary.pdf]

Supplementary Table S1. Factorial ANOVA on arcsine-square-root transformed disease severity data showing consistency between two independent greenhouse trials.

**Factor**	**df**	**F**	**p-value**
**Trial**	1	0.032	0.858
**Thermal treatment**	1	1.753	0.191
**Dose**	3	5.298	**0.003**
**Trial X Thermal treatment**	1	0.026	0.871
**Trial X Dose**	3	0.91	0.442
**Thermal treatment X Dose**	2	1.035	0.362
**Trial X Thermal treatment X Dose**	2	2.216	0.119

Significant effects ($p < 0.05$) are highlighted in bold.
